# Hierarchical coordination of periodic genes in the cell cycle of *Saccharomyces cerevisiae*

**DOI:** 10.1186/1752-0509-3-76

**Published:** 2009-07-20

**Authors:** Frank Emmert-Streib, Matthias Dehmer

**Affiliations:** 1 Computational Biology and Machine Learning, Center for Cancer Research and Cell Biology, School of Medicine, Dentistry and Biomedical Sciences, Queen's University Belfast, 97 Lisburn Road, Belfast, BT9 7BL, UK; 2 Institute of Discrete Mathematics and Geometry, Vienna University of Technology, Wiedner Hauptstrasse 8-10, A-1040 Vienna, Austria; 3 Institute for Bioinformatics and Translational Research, UMIT, Eduard Wallnoefer Zentrum 1, 6060, A-Hall in Tyrol, Austria

## Abstract

**Background:**

Gene networks are a representation of molecular interactions among genes or products thereof and, hence, are forming causal networks. Despite intense studies during the last years most investigations focus so far on inferential methods to reconstruct gene networks from experimental data or on their structural properties, e.g., degree distributions. Their structural analysis to gain functional insights into organizational principles of, e.g., pathways remains so far under appreciated.

**Results:**

In the present paper we analyze cell cycle regulated genes in *S. cerevisiae*. Our analysis is based on the transcriptional regulatory network, representing causal interactions and not just associations or correlations between genes, and a list of known periodic genes. No further data are used. Partitioning the transcriptional regulatory network according to a graph theoretical property leads to a hierarchy in the network and, hence, in the information flow allowing to identify two groups of periodic genes. This reveals a novel conceptual interpretation of the working mechanism of the cell cycle and the genes regulated by this pathway.

**Conclusion:**

Aside from the obtained results for the cell cycle of yeast our approach could be exemplary for the analysis of general pathways by exploiting the rich causal structure of inferred and/or curated gene networks including protein or signaling networks.

## Background

Technological progress during the last decade has generated the innovation of new high-throughput devises in molecular biology that allow to measure the molecular orchestra of genes and products thereof on a genomic scale. Mass data from such experiments, e.g., DNA microarray, yeast two-hybrid or ChIP-chip assay, possess considerable challenges for their statistical data analysis. Due to the fact that a functional understanding of a molecular biological system can only be achieved by studying interactions among gene products network based analysis methods have gained considerable popularity [1-4] because they represent inherently a systems approach [5-8]. The difficulty in analyzing gene networks, e.g., metabolic, signaling or the transcriptional regulatory network [8-11] stems at least partly from the fact that many approaches have been developed outside a biological context [12,13] investigating, e.g., the small-world [14,15] or scale-free [10,16] property of networks. However, so far it is largely unknown how to connect such properties meaningfully to the biological function of a molecular biological system.

In this paper we use the transcriptional regulatory network of yeast to analyze cell cycle regulated genes. More precisely, the major purpose of this article is to shed light on the principal mechanism organizing the cell cycle of *Saccharomyces cerevisiae *by using a novel approach based on the notion of *causal membership*. Our overall approach to analyze cell cycle-regulated genes [17], which are also called *periodic genes *[18], is based on the transcriptional regulatory network of yeast and a list of known genes to be periodically expressed during the cell cycle. No other data are used. This means explicitly that we do not use time series data of, e.g., DNA microarray experiments that would allow to test for a 'periodic behavior' of genes. Hence, our approach is fundamentally different to all other approaches we are aware of studying cell cycle regulated genes of yeast [19-24]. The seeming contradiction to study periodically expressed genes without time series data is resolved quickly by clarifying some terms. First, we want to emphasize that we are interested in genes that are cell cycle regulated. That means genes that *belong to *or *participate in *a certain biological process namely the cell cycle. From a biological point of view this means we are searching for genes that have a biological function that is important for the coordinated initialization and progression of the cell cycle. Hence, statistically we are searching for genes that are *causally *connected to the cell cycle. This is the most precise definition we can give formulated in statistical terms. As we see, logically, there is no need to quantify or qualify further entities including, e.g., the *periodicity *of genes regarding the shape of their signal, to enhance our definition. The *causal membership *of a gene in the biological process *cell cycle *is all we need. Approaches developed so far focus entirely on the periodicity of genes in time series as suggested measure in this respect [19-25]. However, as we explained above it is not imperative to use measures utilizing the periodicity of genes. For this reason we pursue in this paper a novel conceptual path based on the causal membership of genes.

The paper is organized as follows. In the next section we introduce our method and describes the data we use for our analysis. Then we presents numerical results and finish with a discussion and conclusions.

## Methods

High-throughput technologies enable nowadays to tackle the problem of causal inference of gene networks from experimental data [3,26-28] on a genomic scale. Despite the tremendous difficulty of this problem enormous progress has been made during the last years since the seminal work of PEARL et al. [29-31]. In this paper we use a (directed, unweighted) transcriptional regulatory network (TRN) of yeast that has been assembled from different types of high-throughput data [32,33] to ensure that the interactions present in the network correspond to real biologically observable interactions (low number of false positive edges) and, hence, to represent a causal interaction structure. An edge in the TRN connecting, e.g., gene A with gene B implies that there exists a biochemical interaction that has been observed experimentally. For example, gene A might be a transcription factor that is involved in the control of the transcription of gene B. In this paper we study the structure of this causal network to gain functional understanding of the cell cycle of yeast. For clarity, we define now the causal membership of a gene.

**Definition 1 (causal membership) ***The causal membership is an indicator function that indicates if a gene g_*i *_belongs to a certain biological process*.

(1)

For example, it is known that MNN1 (YER001W) [34] is a cell cycle regulated gene. In our terminology this means *I*_*cm*_(cell cycle|MNN1) = 1. Hence, MNN1 is a member of the category cell cycle. In principle, it is possible that one gene is member of more than one category, however, this is not of importance for our investigation because we will focus on just one biological process namely the cell cycle. By introducing definition 1 we want to emphasize the fact that when talking about the biological function of a gene we are interested in the causal involvement of a gene in a certain biological process instead of talking about biochemical properties. If viewed this way it is entirely natural that genes participating, e.g., in the cell cycle can be studied with the help of a causal network representing interactions among these genes. With other words, introducing this level of abstraction helps to see the problem in a different light that would have been overlooked otherwise.

For investigating the organizational structure of genes that are causal members of the biological process cell cycle we use a transcriptional regulatory network *G *and a list of genes known to be periodically expressed. In the following we make the assumption that this transcriptional regulatory network represents all possible causal interactions among genes. No other interactions can occur.

**Assumption 2 ***The transcriptional regulatory network G represents all possible causal interactions among genes*.

We are aware that this assumption is not entirely true because there is also communication among genes involving, e.g., phosphorylation or signaling in general. However, as we will see in the results section, despite the incompleteness of information regarding the consideration of all possible causal interactions, our assumption is sufficient to reveal remarkable results. More information regarding the limitations and possible extensions of our assumption will be given in the discussions section of this article.

**Assumption 3 ***The information among genes can only be transmitted by causal interactions*.

The next assumption makes the purpose of causal interactions clear, their purpose is to transmit information among genes. The information transmission between non adjacent genes is less trivial and far from being fully understood. For this reason we make the following simplified assumption.

**Assumption 4 ***The information between non adjacent genes is transmitted via shortest paths*.

**Figure 1:**
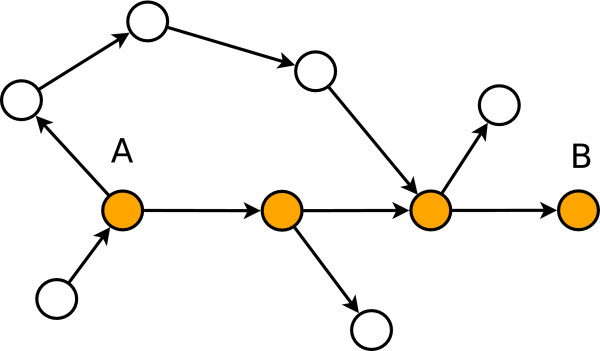
**Information sent from gene A to gene B is transmitted via the shortest path (orange nodes)**.

Here we assume that the significant (molecular) interaction path follows the shortest paths connecting two genes. This assumption is frequently made [10,35,36] when analyzing gene networks. A motivation for this assumption can be given in form of an optimization argument. Non-shortest paths involve more interactions and, hence, consume more energy and time supposing each interaction consumes in average the same amont of engery and involves the same amont of time. For this reason, communication via shortest paths is not only fastest but also cheapest with respect to engery consumption. Fig. 1 visualizes our assumptions in a simplified network. The nodes shown in orange are on a shortest path connecting gene A and B and information from one gene to another can only be transmitted via causal interactions represented by edges in the network.

Finally, we use a property of gene networks to introduce a heterogeneity among genes regarding the transmission of information. It is known that genes and, hence, gene networks, are hierarchically organized [37-39]. In the following we report a property of the TRN that allows to introduce a two-level hierarchy. The transcriptional regulatory network can be partitioned by the presence or absence of cycles connecting genes. In mathematical terms a part of the network that is cyclic is also called a strongly connected component (SCC) [40]. For example, for a SCC containing at least three genes, *A*_*i*_, *A*_*j*_, *A*_*k *_there exists a cycle *A*_*i *_→ ... → *A*_*j *_→ ... → *A*_*k *_→ ... → *A*_*i*_. The dots indicate that there are possibly other genes involved. However, the important thing is that there exists a cycle on which all three genes appear. This observation is important because the presence of a cycle in a network is a necessary condition that truly periodic behavior can be observed because these genes have the ability to interact (activate/inhibit) each other consecutively and, hence, can form a limit cycle [41]. This leads us to the separation of the genes in two classes. The first class consists of genes that belong to the SCC. The genes in the second class do not belong to the SCC. Further the two classes are not equal but the information should flow in one direction namely from *SCC *→ *G/SCC*. The reason is that only genes in the SCC can establish a periodic behavior, as explained above, while genes in *G/SCC *cannot. Based on this classification and hierarchy we state the following assumption.

**Assumption 5 ***The main information flow for the cell cycle in the transcriptional regulatory network connecting periodic genes is organized hierarchically from the SCC to G/SCC*.

From all assumptions we made so far we are now in a position to formulate the hypothesis we will investigate in this paper.

**Hypothesis 6 ***Periodic genes in the SCC of the transcriptional regulatory network of yeast coordinate cell cycle regulated genes via shortest path communication*.

The reason why we formulated this as a hypothesis rather than a theorem is that hypothesis 6 is based on many assumptions (2 – 4) which are difficult to proof theoretically. However, with the help of experimental data (the transcriptional regulatory network and a list of genes known to be periodic) we can falsify our hypothesis 6 numerically. In the results section we will determine all shortest path from periodic genes in SCC to periodic genes in *G/SCC *and investigate the structure of the subnetwork obtained this way. This in turn will provide us with information and insights about our hypothesis.

### Data

For our analysis we use the transcriptional regulatory network (TRN) of *Saccharomyces cerevisiae *[32,33]. This network was assembled from genetic, biochemical and ChIP (chromatin immunoprecipitation)-chip experiments providing above all information about the involvement of transcription factors in the transcription of genes. This network is a directed but unweighted network and each edge represents a biochemical interaction observed experimentally. From this network we extract a weakly connected component (WCC) consisting of 3357 genes and 7230 interactions. The weakly connected component of a network is defined as the directed subnetwork that connects every pair of nodes by at least one directed path [40]. In contrast, the strongly connected component (SCC) is defined as subnetwork that connects each pair of genes in both directions. That means there exists a path connecting, e.g., gene A with gene B but there exists also a path connecting gene B with gene A. The TRN from [32,33] consists of two strongly connected components. One consists of 36 and the other of just 2 genes. When we speak in the following of the SCC of the TRN we speak always about the larger subnetwork also called the giant strongly connected component [42]. The strongly connected component is part of a weakly connected component, *SCC *⊆ *WCC*. We use a list of ZHAO et al. to label genes as 'periodic' [24]. All other genes not labeled 'periodic' are assumed to be 'non-periodic'. ZHAO et al. categorized 260 genes as periodic, however, only 179 periodic genes are in the WCC we use for our analysis. The reason why we restrict our analysis to the WCC is two fold. First, the TRN of yeast is not known entirely. Second, the knowledge of the TRN is not homogeneous but certain regions are better studied than others with respect to the molecular interactions among genes. The WCC can be seen as filtered network providing the highest quality subnetwork of the TRN currently available. Using in addition other parts of the network would increase the noise level considerable and, hence, be counter productive for our analysis.

## Results

We begin our analysis by showing the results we obtain by applying Hypothesis 6 to the transcriptional regulatory network of yeast.

### Organization of the cell cycle

**Figure 2:**
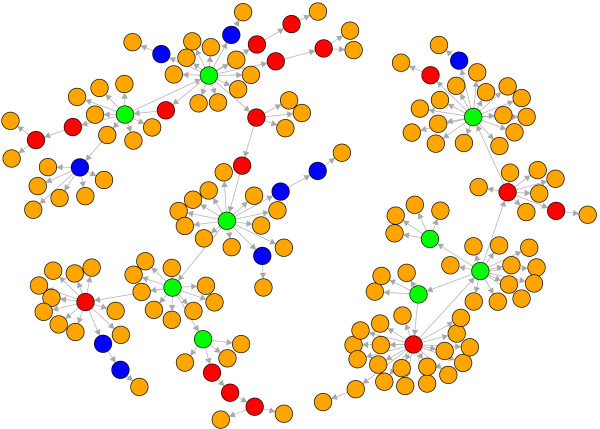
**Subnetwork of the TRN of yeast consisting of 167 genes**. Orange nodes (132): periodic genes according to [24]. Green nodes (9): periodic genes that belong to the SCC. Red nodes (17): Genes that belong to the SCC but are not periodic. Blue nodes (9): Other genes. The connections shown are shortest paths connecting the periodic genes to the strongly connected component. All other connections are omitted.

In Fig. 2 we show a subnetwork of the transcriptional regulatory network that connects all periodic genes in the SCC (in green – a list of these genes is given in table 1) to periodic genes in *G/SCC *(in orange). More precisely, we determined for all periodic genes in *G/SCC *the shortest paths from all periodic genes in the SCC. From these shortest paths the shortest of all has been selected and is displayed in Fig. 2 (if there is more than one we selected a path randomly). The genes shown in red belong to the SCC but are not periodic. The genes in blue are neither in the SCC nor periodic. It is interesting to observe that in this figure there are 141 periodic genes (orange nodes + green nodes). This corresponds to 78% of all 179 periodic genes that are in the WCC we study. The remaining 38 periodic genes do not appear in the figure because there exists no path from the periodic genes in the SCC that would lead to them. Further, there are only 9 additional genes needed (shown in blue) necessary to connect to all 141 periodic genes.

**Table 1:** List of periodic genes in the SCC (green nodes in Fig. 1).

REB1	RAP1	HCM1	YOX1	PHO4	SPT16	ACE2	TOS4	FKH2

**Figure 3:**
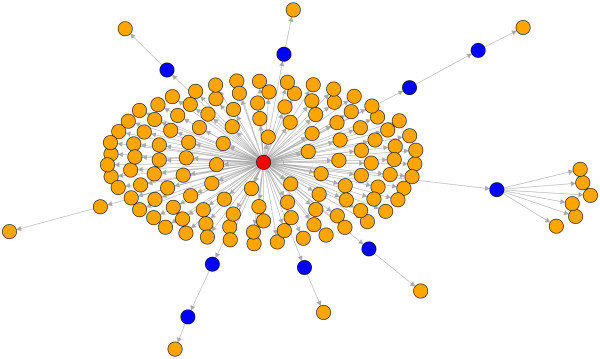
**Subnetwork of the TRN of yeast**. Shown are 141 genes and the strongly connected component represented as one red node. Nodes in orange correspond to periodic genes [24], blue nodes are genes not categorized as periodic. The connections shown are shortest paths connecting the periodic genes to the strongly connected component. All other connections are omitted.

**Figure 4:**
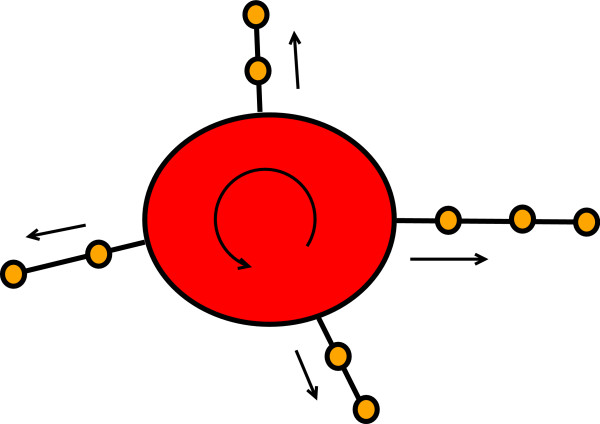
**SCC (red) as pace maker of the cell cycle**. Genes not in the SCC are triggered periodically from genes in the SCC along a cascade involving other periodic genes.

In Fig. 3 we present a centralistic view of the results in Fig. 2. Here all nodes belonging to the SCC (red nodes + green nodes) are represented as one red node. This projection introduces a high order in the network which is now a directed acyclic graph (DAG). Due to the fact that this network is a DAG there are no cycles present connecting genes on closed paths. This is interesting because all genes shown in orange are known to be periodic genes. From Fig. 3 one can clearly see that the majority of periodic genes are connected directly to the SCC. Only very few are connected via paths of length ≥ 1. From the observations in this figure we derive a hypothetical working mechanism of the cell cycle itself visualized in Fig. 4. We propose that there is only a quite small number of genes that are actually connected cyclically. All of these genes have to belong to the SCC because other genes could not be connected cyclically. This small number of genes triggers periodically other genes that are connected to them. In Fig. 4 the big red node represents again the SCC which forms a kind of pace maker because only these genes are connected cyclically. The signal from the SCC is transmitted periodically to the other periodic genes in the system (shown again in orange) via other genes.

### Statistical evaluation of the network structure

Next, we assess statistically our observations made in Fig. 2 and 3. First, we evaluate the number of periodic genes *directly *connected to the SCC by calculating the probability to find more than 127 periodic genes connected to the SCC. Because this gives us a p-value for the observed structure. We do this with a hypergeometric distribution assuming that *k *= 2113 genes (this is the number of genes directly connected to the SCC) are drawn independently from the total set of available genes comprising *m *= 179 periodic and *n *= 3178 non-periodic genes (3357 genes in the WCC minus 179 periodic genes) among which are more than *x *= 127 periodic genes. This gives *P*_*hyp*_(*x *> 127; *m, n, k*) = 0.0083 indicating that such a clustering of periodic genes is unlikely to occur by chance.

**Figure 5:**
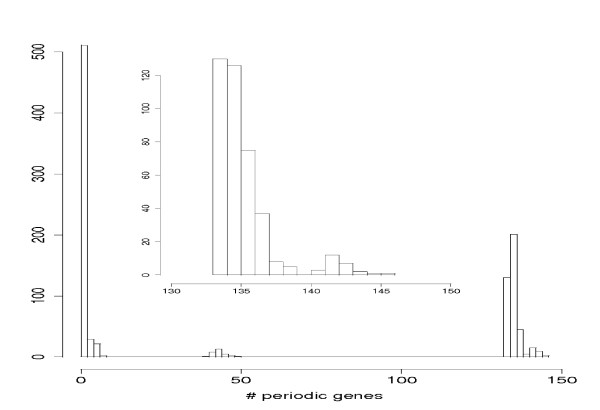
**Histogram of the size of sets consisting of periodic genes that can be connected from an initial set of periodic genes**. The initial set of nine genes was randomly chosen among all 179 periodic genes. The inlay shows a zoom in to the right most region (different binning).

Second, a natural question arising regards the choice of the periodic genes in the SCC as starting point for the shortest paths connecting to other periodic genes outside the SCC. For this reason we select randomly nine genes (because the SCC contains nine periodic genes) among all 179 periodic genes and calculate numerically the size of the periodic gene sets that can be connected this way. From 1000 random selections we find that the largest gene set observed consists of 146 periodic genes. Numerically, we find a p-value of *p *= 0.026 to observe gene sets of size 141 or larger. Interestingly, for all gene sets of size 141 or larger we observe that there is at least one gene of the SCC in the initial set of nine genes. With other words, if in the initial set of genes there is no periodic gene that belongs to the SCC our numerical simulations find only gene sets smaller than 141 genes. This underlines the special role of the SCC in the TRN and supports our hypothesis. This demonstrates that our at first sight ad hoc choice of the SCC is justified by using experimental data in form of the transcriptional regulatory network of yeast [32,33] as well as a list of genes known to be periodically expressed during the cell cycle [24]. In Fig. 5 we show the histogram of our simulations. An interesting observation is that there are roughly three regions representing three different sizes of periodic gene sets. This can be explained with the help of Fig. 2. There one can see that the leaf nodes are periodic genes (orange nodes). As it turns out trying to find paths from these leaf nodes to other periodic genes is in most cases not possible because there is no such path in the transcriptional regulatory network. If one would choose such periodic genes (leafe nodes) as starting point to find shortest paths to all other periodic genes (as we did in our randomized simulations as explained above) one will not be able to connect many periodic genes. This explains the left most region in the histogram in Fig. 5. Another look to Fig. 2 reveals that there are two separated subnetworks that are not connected by any path. This explains the middle region in Fig. 5 because in this case periodic genes have been chosen as starting point that are all on the same island corresponding to a subnetwork of the transcriptional regulatory network from which there are no paths connecting to periodic genes outside this region. Only if there are periodic genes in the initial set of starting genes from both islands a high number of periodic genes can be connected (right most region in Fig. 5).

From these statistical evaluations we conclude the following: First, the high number of periodic genes connected to the SCC cannot be explained by chance. Second, selecting periodic genes from the SCC as starting point for the determination of shortest paths connecting to other periodic genes is absolutely necessary to reach such a high number of periodic genes (141) in the WCC.

### Evaluating non-periodic genes

Finally, we take a closer look to the nine genes in Fig. 2 and 3 (shown in blue) that are non-periodic genes according to the gene list we use as reference [24]. Because so far there is no general agreement in the literature regarding all periodic genes of the cell cycle we use three more lists obtained from genome scale experiments. We use information from Johnansson et al. [43], de Lichtenberg et al. [18] and Cyclebase [34] and find that two of the nine non-periodic genes are actually declared periodic by CYCLEBASE and also ranked quite low in the other two gene lists from Johnansson et al. and de Lichtenberg et al.. Table 2 provides the information of the ranks of these two genes. This leaves us with merely seven non-periodic genes in Fig. 2 and 3 for which so far insufficient information is available to be able to declare them as periodic.

**Table 2:** Genes declared to be periodic by Cyclebase (per).

gene	Johnansson et al.	de Lichtenberg et al.	Cyclebase
TEC1	239	104	319 (per)
SWI5	109	79	124 (per)

The numbers in the second, third and fourth column correspond to the ranking (low values are more likely to be periodic) according to Johnansson et al. [43], de Lichtenberg et al. [18] and Cyclebase [34].

The interpretation from these results is that the application of hypothesis 6 to the transcriptional regulatory network (TRN) of *Saccharomyces cerevisiae *[32,33] and a list of periodic genes reveals that our hypothesis is consistent with the data capable of explaining nearly 80% of all periodic genes in the WCC. We found that there are only seven genes inconsistent with hypothesis 6 assuming that the reference list of periodic genes is absolutely true (no false positives, no false negatives).

## Conclusion

In this paper we raised a hypothesis regarding the organizational structure of the cell cycle of *S. cerevisiae*. To formulate our hypothesis we partitioned the set of periodic genes in two groups according to a graph theoretical property leading to a hierarchy in the transcriptional regulatory network from the SCC to *G/SCC*. We hypothesized that periodic genes in the SCC coordinate cell cycle regulated genes via shortest paths. We presented numerical results testing our hypothesis by using the transcriptional regulatory network from [32,33] and a list of genes known to be periodically expressed [24].

Our numerical results demonstrate that by applying our hypothesis to the data (transcriptional regulatory network and the reference list of genes known to be periodically expressed) we find a subnetwork of the overall transcriptional regulatory network connecting almost 80% of all periodic genes in the WCC. A statistical evaluation of the observed network structure revealed that, first, the high number of periodic genes connected to the SCC cannot be explained by chance. Second, selecting periodic genes from the SCC as starting point for the determination of shortest paths connecting to other periodic genes is absolutely necessary to reach such a high number of connected periodic genes (141). This indicates that our at first sight ad hoc hypothesis reflects structural information manifested by the transcriptional regulatory network. Further, we interpret our results conceptionally in a way that the SCC forms a pacemaker of the cell cycle because only genes in the SCC can form cycles (closed paths) and, hence, only these genes can be truly periodic mathematically [41]. To our knowledge the SCC of the transcriptional regulatory network has so far not been interpreted as pacemaker of the cell cycle of yeast.

We based our hypothesis on the transcriptional regulatory network of yeast assuming that this network represents all important causal interactions among genes that might play a crucial role for the information transmission of the system. It is clear that this is a simplification ignoring signaling among genes, e.g., via phosphorylation, to name just one additional effect. For this reason there is another way to interpret our results: How much information regarding the organizational structure of the cell cycle is contributed by the transcriptional regulatory network only. As demonstrated by our numerical results the transcriptional regulatory network seems to make a remarkable high contribution to this because otherwise our hypothesis would not span nearly 80% of the periodic genes in the WCC. This is an interesting result for itself. Furthermore, it would be interesting to see if using additional networks, e.g., the signaling network, helps to improve our results considerably. Also, it is clear that the transcriptional regulatory network we used for our analysis is not complete (false negative edges) nor absolutely correct (false positive edges). For this reason we filtered the overall network using only the WCC to extract a high quality subnetwork. It will be interesting to repeat our analysis in a couple of years using a revised version of the transcriptional regulatory network to see if this network leads to an improvement of our results. Due to the fact that the used network has been assembled from different sources of high-throughput data [32,33] the probability of false positive edges is expected to be quite low. This means that further experimental results are unlikely to reduce the quality of our results. In contrast, there are certainly quite a few interactions among genes (edges in the network) that are currently absent in the used network (false negative edges). These edges can only lead to an improvement of our results because additional edges can only lead to new paths but not destroy existing ones.

Based on our observations one might speculate that our hypothesis may not only hold for the cell cycle of *S. cerevisiae *but also for the cell cycle of other organisms. If this would be true then the organizational structure would be evolutionary conserved among organisms. This would provide another important feature for evolutionary biologists next to, e.g., the conservation of protein sequences and structures as well as network motifs, allowing to assess homology on a systems level comprising a functional biological pathway. Further, our concept of *causal membership *to a biological process may also be extendable to other biological processes than the cell cycle as well as other organisms. Our approach does not utilize information that specifically holds only for the problem studied in this paper. For example, biological processes like apoptosis, cellular differentiation or cell signaling could be studied. It would also be interesting to use our approach in the context of complex diseases like cancer to study pathological modifications of such biological pathways. This seems to be feasible provided the causal network used for the analysis contains sufficient information covering essential aspects of the underlying molecular interactions. In this respect it might be beneficial to combine the transcriptional regulatory network with the signaling or protein network. Not only because this may lead to an increased performance but also to learn about differences of the information encoded in these networks. The latter point could contribute to enhance our understanding of the integration of different types of gene networks which has not been received much attention so far.

Generally, we want to remark that the property *cyclicity *of a network, used in this paper to define the SCC, has been already used previously to meaningfully separate molecular networks [44] but in the context to identify structural domains of proteins.

We are of the opinion that approaches similar to ours [33,45], exploiting the causal network structure of a gene network, will gain rapidly more attention because with the availability of estimation methods to infer causal network structures from high-throughput data [26,28] the interest will gradually shift towards their analysis. The reason therefor is that gene networks are certainly of interest themselves, however, more interesting is it to use them to disclose *functional *biological information. If the overall process of the yeast cell cycle follows our hypothesis will be subject to further studies. However, the conceptual structure as revealed by our simple organization of the data, in form of the transcriptional regulatory network, could be exemplary for general studies aiming not only to identify 'important' genes but also to shed light on working principles.

## Authors' contributions

Both authors contributed to all parts of the article.
